# Effect of antibiotic drug use on outcome and therapy-related toxicity in patients with glioblastoma—A retrospective cohort study

**DOI:** 10.1093/noajnl/vdae170

**Published:** 2024-10-04

**Authors:** Linda Götz, Tananeh Ansafi, Michael Gerken, Monika Klinkhammer-Schalke, Anna Fischl, Markus J Riemenschneider, Martin Proescholdt, Elisabeth Bumes, Oliver Kölbl, Nils Ole Schmidt, Ralf Linker, Peter Hau, Tareq M Haedenkamp

**Affiliations:** Department of Neurology and Wilhelm Sander-NeuroOncology Unit, Regensburg University Hospital, Regensburg, Germany; Department of Neurology and Wilhelm Sander-NeuroOncology Unit, Regensburg University Hospital, Regensburg, Germany; Center for Quality Assurance and Health Services Research, University of Regensburg, Regensburg, Germany; Center for Quality Assurance and Health Services Research, University of Regensburg, Regensburg, Germany; Department of Neurology and Wilhelm Sander-NeuroOncology Unit, Regensburg University Hospital, Regensburg, Germany; Department of Neuropathology, Regensburg University Hospital, Regensburg, Germany; Department of Neurosurgery, Regensburg University Hospital, Regensburg, Germany; Department of Neurology and Wilhelm Sander-NeuroOncology Unit, Regensburg University Hospital, Regensburg, Germany; Department for Radiotherapy, Regensburg University Hospital, Regensburg, Germany; Department of Neurosurgery, Regensburg University Hospital, Regensburg, Germany; Department of Neurology and Wilhelm Sander-NeuroOncology Unit, Regensburg University Hospital, Regensburg, Germany; Department of Neurology and Wilhelm Sander-NeuroOncology Unit, Regensburg University Hospital, Regensburg, Germany; Department of Neurology and Wilhelm Sander-NeuroOncology Unit, Regensburg University Hospital, Regensburg, Germany

**Keywords:** antibiotics, glioblastoma, intestinal microbiome, survival, therapy-related toxicity

## Abstract

**Background:**

Glioblastoma (GB) is the most frequent malignant brain tumor and has a dismal prognosis. In other cancers, antibiotic use has been associated with severity of chemotherapy-induced toxicity and outcome. We investigated if these mechanisms are also involved in GB.

**Methods:**

We selected a cohort of 78 GB patients who received combined radiochemotherapy. We investigated if exposure to prediagnostic antibiotic use is associated with clinical side effects and laboratory changes during adjuvant therapy as well as overall survival (OS) and progression-free survival (PFS) using chi-square test, binary logistic regression, Kaplan–Meyer analysis, and multivariable Cox regression.

**Results:**

Seventeen patients (21.8%) received at least one course of prediagnostic antibiotics and 61 (78.2%) received no antibiotics. We found a higher incidence of loss of appetite (23.5% vs. 4.9%; *P* = .018) and myelosuppression (41.2% vs. 18.0%; *P* = .045) in the antibiotic group. Multivariable logistic regression analysis revealed antibiotics to be a predictor for nausea (OR = 6.94, 95% CI: 1.09–44.30; *P* = .041) and myelosuppression (OR = 9.75, 95% CI: 1.55–61.18; *P* = .015). Furthermore, lymphocytopenia was more frequent in the antibiotic group (90.0% vs. 56.1%, *P* = .033). There were no significant differences in OS (*P* = .404) and PFS (*P* = .844). Multivariable Cox regression showed a trend toward shorter survival time (*P* = .089) in the antibiotic group.

**Conclusions:**

Our study suggests that antibiotic use affects symptoms and lab values in GB patients. Larger prospective studies are required to investigate if prediagnostic antibiotic use could be a prognostic factor in GB patients.

**Key Points**

- Antibiotic treatment may have an impact on symptoms and laboratory results during adjuvant therapy in patients with glioblastoma.
- Patients with prediagnostic antibiotic use show no difference in OS and PFS when compared to patients without antibiotic treatment.

**Importance of the Study**

Administration of antibiotics has been shown to be associated with toxicity and outcome of various cancer therapies. However, much less information is available for patients with glioblastoma (GB). This study provides valuable insights into the potential impact of prediagnostic antibiotic use on clinical outcomes and laboratory changes in GB patients. It reveals a significant association between antibiotic use and increased incidence of side effects such as loss of appetite and nausea. The study also suggests a possible link between antibiotic use and lymphocytopenia. While there were no significant differences in overall survival and progression-free survival, a trend towards shorter survival time in the antibiotic group was observed. These findings could have important implications for the management of GB patients and warrant further investigation in larger prospective studies.

Glioblastoma (GB), IDHwt, CNS WHO grade 4 develops in the CNS and is considered the most common primary malignant parenchymal neoplasm in adults in the Western world, making up around 50.9 % of malignant brain tumors.^1^ Due to multifactorial reasons such as ethnicity, socioeconomic status, healthcare provision, and epidemiological recordings, the incidence varies.^1^ GB has a median survival time of 20.9 months despite therapy with resection, radiochemotherapy, and tumor-treating fields. Therefore, this lethal diagnosis is an important subject of research.^2^

Several factors have been detected to be prognostic, including age, gender, extent of resection, performance score, methylation of the *MGMT* promoter, and temporal muscle thickness.^3–5^ Other factors such as density of treatment^5^ and prevalence of side effects^6^ may also influence prognosis. In the context of standard-of-care simultaneous radiochemotherapy following the Stupp regimen, multiple side effects have been described.^7–9^ These include, among others, nausea, vomiting, bone marrow suppression, opportunistic infections, fatigue, and liver dysfunction.^7–9^ In addition, the use of alkylating agents often leads to hematological changes in laboratory values such as anemia, leukopenia, and thrombocytopenia.^7–9^ In recent publications, prolonged bone marrow suppression has been accused of being connected to inferior survival times.^10^ These and other therapy-induced side effects can differ depending on dose, biodistribution, drug metabolism, mechanisms of action, and combined effects of pharmaceuticals used in multimodal regimens.^11^

In recent years, there has been increasing evidence that the gastrointestinal microbiome influences not only the development of different types of cancer but also plays a pivotal role in the efficacy of cytostatic drugs.^12^ Suggested mechanisms include modifications of drug metabolism^13,14^ or regulation of immune responses.^12,15^

Of note, modulation of the gastrointestinal microbiome can occur due to the use of probiotics or antibiotics, dietary interventions, genetics, lifestyle, medication, cancer itself, and infections.^16,17^ Several microbiota have been found to influence the effect of chemotherapeutic agents and their therapy-limiting side effects via their enzymatic function.^12^ The risk of serious side effects was increased in patients who were treated with antibiotics^18^ and the intestinal microbiota composition was shown to be associated with toxicity.^19^ Another study showed an increased risk of hematologic and gastrointestinal side effects after administration of the antibiotic gemcitabine.^20^

Recently, several studies in various cancers also showed that antibiotic drug use modulates the efficacy of cytostatic drugs. In humans, a detrimental influence of antibiotic treatment on the outcome of lymphoma patients^21,22^ has been observed. The efficacy of immune checkpoint inhibitors is impaired in patients who received antibiotic drugs before immune therapy.^18,23–25^ The impact of antibiotics on glioma growth was also investigated in mice previously.^26–28^ There are indirect findings in vitro suggesting that antibiotics promote tumor growth via enhancement of vasculogenesis.^28^ Animals that received antibiotics showed strongly increased tumor growth, presumably due to the reduction of cytotoxic natural killer cells, an altered composition or reduced diversity of the intestinal microbiota, and microglial alterations towards a protumoral and immunosuppressive phenotype.^26^ Another animal study with a glioma xenograft model revealed differences in the gut microbiota of mice that were sensitive to temozolomide compared to those that were not.^27^ Modification of microbiota by antibiotic treatment could attenuate temozolomide efficacy.^27^ Lastly, animal models indicate that the use of antibiotics might lead to reduced efficacy of agents like 5-fluorouracil,^29^ platinum,^30^ or cyclophosphamide.^15^

In addition, epidemiological studies showed a slightly increased risk of glioma after antibiotic use.^31,32^ These observations point in the direction of possible effects that may occur during glioma development, in contrast to the aforementioned effects, that may occur in full-blown tumors.

In view of the unanswered questions detailed above, we hypothesized that prediagnostic administration of antibiotic drugs might affect the toxicity of chemotherapy, lab values, progression, and overall survival in patients with GB. We therefore performed a retrospective study in a homogeneous cohort of patients with GB to answer the question if the use of antibiotic drugs may be associated with clinical and hematological side effects and long-term prognosis in these patients.

## Methods

### Study Cohort

This study was conducted as a retrospective longitudinal study with prospective data collection. All patients with GB who were treated from January 2010 to December 2019 at the certified Neuro-oncology Center Regensburg were eligible for the study. Contact data of patients and general practitioners were obtained from the cancer registry of the University Hospital Regensburg. All patients or, in case of death, their proxies were contacted to obtain written informed consent for the study. Before study initiation, approval from the local ethics committee of the University of Regensburg was obtained (Ethics vote: 20-1809-101).

### Data Acquisition

We used newly developed questionnaires to query data on past antibiotic use from GPs. The questionnaires were verified for clarity and completeness by 2 independent internal controls. Data on histology and genetics of the tumor, demographics like prognostic and survival data of patients that included age, sex, body mass index (BMI), previous independent tumor disease, *MGMT* promoter methylation status, *IDH* mutational status, tumor recurrence, Karnofsky performance score and extent of resection were collected.

In addition, clinical information such as therapy regimen and its duration, side effects of radiochemotherapy over the whole treatment period such as loss of appetite, nausea, vomiting, diarrhea, obstipation, skin reactions, dizziness, headache, fatigue, alopecia, BMI and comorbidities were collected.

We also collected information on the number, frequency, duration, drug class, and additional medication for antibiotic use administered up to 12 months before diagnosis. Since all patients received perioperative antibiotic prophylaxis, we defined antibiotic drug exposure as occurring only before the time of diagnosis. In addition, laboratory values were extracted from the local laboratory data acquisition systems of the University Hospital Regensburg and affiliated hospitals. Lab values were extended by patient file recordings of laboratory parameters from GPs. As for laboratory values, we not only summarized parameters over the entire treatment period but also predefined 4-time points after diagnosis, namely week 1, week 12, week 20, and week 28 +/−2 after resection or biopsy, for statistical lab evaluations. We collected laboratory data on C-reactive protein (CRP), leukocytes, lymphocytes granulocytes, thrombocytes, and hemoglobin. Missing values were documented as blanks.

After data acquisition, the data were pseudonymized in 2 steps and stored locally in a data-secured manner that is in accordance with EU and local data protection regulations. A data protection concept is filed.

### Clinical Endpoints

Clinical data, blood counts from the laboratory systems, and side effects were filed. Due to the lack of data on the severity of clinical side effects, it was not possible to classify them according to CTC grades. Blood counts were operationalized according to Common Terminology Criteria for Adverse Events v 5.0 (CTC) at the predefined timepoints (Week 1, 12, 20, and 28 +/−2 after resection) during the adjuvant radiochemotherapy treatment period, with the goal to file toxicity of chemotherapy. Laboratory values were divided into CTC grades 1–4 according to common cutoffs (accessible under https://ctep.cancer.gov/protocolDevelopment/electronic_applications/ctc.htm). All values greater than or equal to CTC grade 3 were considered as relevant toxicity of antibiotics in comparison to the non-antibiotic group.

### Statistical Analyses

All analyses were carried out using IBM SPSS Statistics, version 29. For metric variables, the mean, median, standard deviation, minimum, and maximum were specified. The Pearson chi-square test was used to test the independence of categorical variables and the administration of antibiotics. Comparisons of means were made using the Student *t*-test and Mann–Whitney U-test. Multivariable binary logistic regression analyses were performed by adjusting for the potentially confounding variables age, sex, BMI, previous independent tumor disease and *MGMT* promotor methylation status, *IDH* mutational status, tumor recurrence, Karnofsky performance score, and extent of resection. We selected these variables a priori due to their known or assumed impact on the outcome or because of their potential to influence the likelihood of antibiotic prescription.

The association between antibiotic use to side effects and changes in laboratory values were analyzed using univariable and multivariable binary logistic regression analysis. All *P*-values <.05 were considered statistically significant.

Kaplan–Meier analyses and multivariable Cox regression analyses were performed to estimate overall survival and progression-free survival (PFS). The log-rank test was used to compare survival curves.

## Results

### Study Cohort

**Figure 1.**
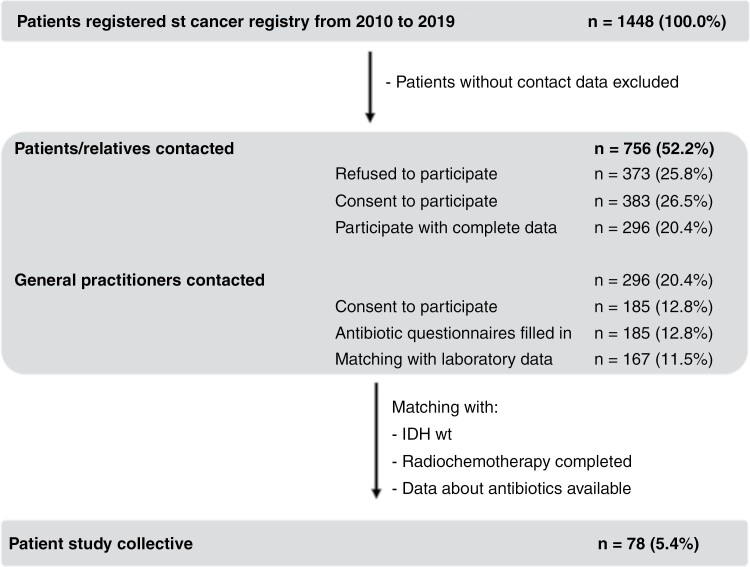
Flow chart of patients with prospective data collection.

Our original cohort consisted of 1448 glioma patients from the local cancer registry databases, who were treated at the certified Neuro-oncology Center in Regensburg between 2010 and 2019. By excluding pediatric patients and patients who were externally treated as well as patients without or with insufficient contact data, the initial cohort was reduced to 756 patients (52.2%). These patients and their relatives were contacted by telephone to ask for their participation. Of these, 296 patients or relatives (20.4%) provided written informed consent via post. The GPs of all patients who gave informed consent were called, and 185 (12.8%) of these agreed to provide the requested information. In this analysis, only patients with GB, IDHwt (CNS WHO grade 4) who received treatment according to the Stupp regimen,^7^ further on being referred to as standard therapy, and who provided information about prediagnostic antibiotic use were included. All 78 patients (5.4%) for whom complete information was available were included in our final analysis (Figure 1).

The median age in our cohort was 59 years (range 30–79 years). There were more males than females (62.8% vs. 37.2%), and patients with prediagnostic antibiotics were younger and more likely to be aged between 50 and 59 years (64.7%). Of the 78 patients in the final analysis, 17 (21.8%) received one or more antibiotic drugs within 12 months before the time of GB diagnosis, while 61 (78.2%) were not treated with antibiotics. 10 patients (12.8%) received only one antibiotic drug, 4 (5.1%) received 2 courses and 3 (3.8%) had 3 antibiotic treatments (Supplementary Table S1). Six patients (7.6%) were treated with inhibitors of cell wall synthesis, 4 (5.1%) with interactors of bacterial DNA or RNA, and 7 (9.0%) with inhibitors of bacterial protein synthesis. See Supplementary Table S2 for details on specific antibiotic substances.

Patients who received antibiotics before diagnosis tended to be overweight with a BMI of 25–29 kg/m² (47.1%). In total, 82.4% of patients who received prediagnostic antibiotics had a KPS of 90 or more, compared to 59.0% who did not. None of these differences were statistically significant (Table 1).

**Table 1.** Patient Characteristics of the Study Cohort Dependent on Prediagnostic Use of Antibiotics Compared to No Use

Prediagnostic antibiotic use		Yes		No		Total		
		*n*	%	*n*	%	*n*		%
	17	21.8	61	78.2	78		100.0
Prediagnostic antibiotics in relation to prognostic factors		yes		no		total		Chi^2^
		*n*	%	*n*	%	*n*	%	*P*
Sex	female	6	35.3	23	37.7	29	37.2	.856
	male	11	64.7	38	62.3	49	62.8	
Age groups	<50.0	1	5.9	11	18.0	12	15.4	.112
	50.0–59.9	11	64.7	20	32.8	31	39.7	
	60.0–69.9	4	23.5	21	34.4	25	32.1	
	≥70.0	1	5.9	9	14.8	10	12.8	
BMI (kg/m²)	<25.0	5	29.4	18	29.5	23	29.5	.775
	25.0–29.9	8	47.1	22	36.1	30	38.5	
	≥30.0	3	17.6	13	21.3	16	20.5	
	unknown	1	5.9	8	13.1	9	11.5	
*IDH*	mutation	0	0.0	0	0.0	0	0.0	
	wildtype	17	100.0	61	100.0	78	100.0	
*MGMT*	not methylated	8	47.1	26	42.6	34	43.6	.676
	methylated	7	41.2	31	50.8	38	48.7	
	unknown	2	11.8	4	6.6	6	7.7	
Extent of resection	Complete resection	6	35.3	27	44.3	33	42.3	.557
	Biopsy/partial resection	11	64.7	32	52.5	43	55.1	
	unknown	0	0.0	2	3.3	2	2.6	
Karnofsky score	<70	0	0.0	4	6.6	4	5.1	.624
	70	1	5.9	7	11.5	8	10.3	
	80	1	5.9	8	13.1	9	11.5	
	90	8	47.1	20	32.8	28	35.9	
	100	6	35.3	16	26.2	22	28.2	
	unknown	1	5.9	6	9.8	7	9.0	
Previous other tumor disease	yes	3	17.6	4	6.6	7	9.0	.157
	no	14	82.4	57	93.4	71	91.0	

### Occurrence of Side Effects Anytime During the Treatment Period

First, we investigated whether the occurrence of side effects, as reported in clinical records, during the entire treatment period differs between patients who were treated with antibiotics and those who were not (Table 2). Overall, patients with at least one prediagnostic antibiotic administration tended to show an increased rate of side effects (at least one side-effect: 88.2%) compared to patients without antibiotic administration (68.9%; *P* = .111). When individual side effects were analyzed, a significantly higher rate of loss of appetite (23.5% vs. 4.9%; *P* = .018) and myelosuppression (41.2% vs. 18.0%; *P* = .045) was shown in patients with prediagnostic antibiotic administration. In addition, we observed a strong trend for a higher occurrence of nausea (52.9% vs. 27.9%; *P* = .052) and obstipation (5.9% vs. 0.0%; *P* = .057) in patients with prior antibiotic use. Regarding all other side effects, no significant differences were observed (Table 2).

**Table 2.** Adverse Events in Patients With Prediagnostic Antibiotic Use Compared to No Use According to CTC AE 5.0

Prediagnostic antibiotic use						Binary logistic regression			
	Yes (*n* = 17)		No (*n* = 61)		Chi^2^ *P*	univariable		multivariable^***^	
	*n*	%	*n*	%		*P*	OR (95% CI)	*P*	OR (95% CI)
Any side effect	15	88.2	42	68.9	.111	.128	3.39 (0.71–16.34)	.025	20.12 (1.46–277.00)
Myelosuppression	7	41.2	11	18.0	.045	.052	3.18 (0.99–10.21)	.015	9.75 (1.55–61.18)
Inflammation	0	0.0	4	6.6	.278	.278	*0.37 (0.02–7.12)	^**^-	^**^-
Skin reaction	0	0.0	7	11.5	.143	.143	*0.21 (0.01–3.82)	^**^-	^**^-
Nausea	9	52.9	17	27.9	.052	.058	2.91 (0.97–8.79)	.041	6.94 (1.09–44.30)
Vomiting	1	5.9	8	13.1	.409	.422	0.41 (0.05–3.56)	.318	0.17 (0.01–5.68)
Diarrhea	1	5.9	2	3.3	.622	0.626	1.84 (0.16–21.65)	^**^-	^**^-
Constipation	1	5.9	0	0.0	.057	.057	*11.18 (0.44–287.35)	^**^-	^**^-
Loss of appetite	4	23.5	3	4.9	.018	.030	5.95 (1.19–29.86)	.630	0.39 (0.01–18.59)
Dizziness	2	11.8	10	16.4	.640	.642	0.68 (0.13–3.45)	.869	0.82 (0.08–4.26)
Headache	2	11.8	10	16.4	.640	.642	0.68 (0.13–3.45)	.898	0.86 (0.08–9.55)
Fatigue	10	58.8	31	50.8	.559	.560	1.38 (0.47–4.11)	.445	1.74 (0.42–7.13)
Alopecia	4	23.5	9	14.8	.391	.395	1.78 (0.47–0.69)	.206	3.71 (0.49–28.41)

^*^Haldane correction (value 0.5).

^**^low case numbers.

^***^Adjustment for the following variables: sex, age, BMI, MGMT, Karnofsky score at time of resection, extent of resection, previous independent tumor disease.

In our univariable regression analysis, prediagnostic antibiotic use was a significant predictor of appetite loss (OR = 5.95, 95% CI: 1.19–29.86; *P* = .030) during the course of therapy. All other variables partly showed a trend, namely total side effects (OR = 3.39, 95% CI: 0.70–16.33; *P* = .128), nausea (OR = 2.91, 95% CI: 0.96–8.78; *P* = .058), constipation (OR = 11.18, 95% CI: 0.44–287.35; *P* = .057), myelosuppression (OR = 3.18, 95% CI: 0.99–10.20; *P* = .052), and skin reaction (OR = 0.21, 95 % CI: 0.0–3.82; *P* = .143), but were not statistically significant. In our multivariable regression model, prediagnostic antibiotic use was found to be a significant predictor of overall side effects (OR = 20.12, 95% CI: 1.46–277.00; *P* = .025), nausea (OR = 6.94, 95% CI: 1.09–44.30; *P* = .041), and myelosuppression (OR = 9.75, 95% CI: 1.55–61.18; *P* = .015).

### Sensitivity Analyses

To evaluate the robustness of our results, we conducted 2 sensitivity analyses.

In our main analysis, potential confounders in the multivariable model were selected a priori. In the first sensitivity analysis, we included only variables that showed differences in distribution with a Chi-square *P*-value lower than .200. These variables were age at diagnosis (*P* = .112) and previous tumor (*P* = .157). Overall, the findings are consistent with the results from the multivariable analysis that included all variables, as shown in Table 2, with the exception that the risk of loss of appetite remains significant in the multivariable model (Supplementary Table S3).

In the second sensitivity analysis, we included 2 cases where antibiotics were used after diagnosis but before the start of radiochemotherapy, without prior antibiotic use before diagnosis. This analysis largely confirms the original results in Table 2, except for nausea, which is now marginally non-significant (*P* = .061). However, “any side effects” (*P* = .019) and “myelosuppression” (*P* = .010) remain significant in the multivariable regression analysis (Supplementary Table S4).

### Laboratory Value Changes at Predefined Timepoints

Next, we investigated the association between antibiotic use and laboratory alterations at predefined timepoints (weeks 1, 12, 20, and 28; Table 3). Looking at laboratory value alterations in the first week after initial diagnosis, 60.0% of patients in the antibiotic group had reduced lymphocyte parameters (CTC 3–4). In the comparison group, the frequency of lymphopenia was only 18.8%, with the vast majority (81.3%) showing only moderately or no reduced lymphocytes according to CTC grade 0–2 (*P* = .075; Table 3). Changes in lymphocytes from the univariable regression analyses still showed a trend but were not significant in the multivariable model (OR = 6.5, 95% CI: 0.73–57.82; *P* = .093; Supplementary Table S4).

**Table 3.** CRP Und Lymphocyte Changes in Patients With Prediagnostic Antibiotic Use Compared to No Use in Weeks 1, 12, 20, 28 +/− 2 After Time of Diagnosis

Laboratory alterations		Prediagnostic antibiotic use					Binary logistic regression			
		Yes		*N*o		*P*	Univariable		Multivariable^**^	
		*n*	%	*n*	%		*P*	OR (95% CI)	*P*	OR (95% CI)
Week 1										
CRP (mg/l)	<5.0 ≥5.0	4 3	57.1 42.9	26 11	70.3 29.7	.494	.104	3.06 (0.80–11.76)	.021	20.858 (1.57–277.00)
Lymphocytes	CTC 0–2 CTC 3–4	2 3	40.0 60.0	13 3	81.3 18.8	.075	.093	6.50 (0.73–57.83)	—	^*^-
Week 12										
CRP (mg/l)	<5.0 ≥5.0	9 0	100.0 0	22 4	84.6 15.5	.211	.209	0.24 (0.03–2.20)	.16	0.06 (0.00–3.08)
Lymphocytes	CTC 0–2 CTC 3–4	4 5	44.4 55.5	13 14	48.1 51.9	.847	.847	1.61 (0.26–5.29)	.36	2.93 (0.29–29.94)
Week 20										
CRP (mg/l)	<5.0 ≥5.0	3 2	60.0 40.0	14 2	87.5 12.5	.172	.137	4.67 (0.61–35.49)	—	^*^-
Lymphocytes	CTC 0–2 CTC 3–4	2 5	28.6 71.4	5 11	31.3 68.8	.898	.089	1.14 (0.16–8.00)	—	^*^-
Week 28										
CRP (mg/l)	<5.0 ≥5.0	4 2	66.7 33.3	17 1	94.4 5.6	.075	.464	2.13 (0.28–15.97)	—	^*^-
Lymphocytes	CTC 0–2 CTC 3–4	2 6	25.0 75.0	7 14	33.3 66.7	.665	0.666	1.50 (0.24–9.44)	—	^*^-

^*^low case numbers.

^**^Adjustment for the following variables: sex, age, BMI, MGMT, Karnofsky score at time of resection, extent of resection, previous independent tumor disease.

CRP levels in week 1 were increased in 42.9% of patients in the group treated with antibiotics compared to 29.7% without antibiotics. After adjustment for possible confounders, we found significantly increased CRP values in the antibiotic group (OR = 20.858, 95% CI: 1.57–277.00; *P* = .021). At week 28, 33.3% of patients in the antibiotic group had an elevated CRP (*P* = .075; Table 3). No difference was observed in the logistic regression analysis (Supplementary Table S4).

At week 1, a trend towards decreased lymphocyte levels was observed in patients with previous use of antibiotics (*P* = .075). Results of univariable analysis in week 1 as well as in week 20 followed these trends. However, no difference was observed at weeks 12 and 28 (Table 3).

### Anytime Occurrence of Laboratory Changes During the Entire Treatment Period

We next analyzed the association between antibiotic use and laboratory value alterations over the entire treatment period. Over the course of their disease, 90.0% of patients with prediagnostic antibiotics had decreased lymphocytes compared to 56.1% in the group without antibiotic therapy which was statistically significant (*P* = .033; Table 4).

**Table 4.** Hematologic Toxicity Parameters Depending on Prediagnostic Antibiotic Use Versus No Use During the Entire Observation Period Comparing CTC AE 5.0 Grade 3–4 versus 1–2

Laboratory alterations	CTC	Prediagnostic antibiotic use				
		Yes		No		*P*
		*n*	%	*n*	%	
Leukocytes (mio/mL)	0–2	15	93.8	50	92.6	.875
	3–4	1	6.3	4	7.4	
Lymphocytes (mio/mL)	0–2	1	9.1	18	43.9	.033
	3–4	10	90.9	23	56.1	
Granulocytes (mio/mL)	0–2	10	90.9	36	87.8	.775
	3–4	1	9.1	5	12.2	
Thrombocytes (mio/mL)	0–2	14	87.5	51	94.4	.343
	3–4	2	12.5	3	5.6	
Hemoglobin (g/100 mL)	0–2	16	100.0	51	94.4	.335
	3–4	0	0.0	3	5.6	
CRP (mg/l)	<5.0	12	70.6	43	70.5	.994
	≥5.0	5	29.4	18	29.5	

Based on the results of the chi-square test, a binary logistic regression analysis was carried out for the change in lymphocyte values over the entire treatment period. The analysis showed a trend for a reduction in total lymphocytes with prediagnostic antibiotic administration (OR = 7.83, 95% CI: 0.92–66.93; *P* = .060). This finding was significant in the multivariable regression analysis (OR = 81.68, 95% CI: 2.02–3298.01; *P* = .020).

Results for differences regarding total leucocytes, granulocytes, thrombocytes, hemoglobin, and CRP were not statistically significant (Table 4).

Comparisons of means for blood values revealed higher CRP and lower numbers of leukocytes, thrombocytes, lymphocytes, and granulocytes in patients who used antibiotics compared to those who did not. However, all differences in means from the Student’s *t*-test and multiple regression analyses were not statistically significant (Supplementary Table S5).

### Survival Analysis

Finally, we examined if antibiotic use before diagnosis was associated with long-term outcomes in GB. The median follow-up time was 3.8 years in our cohort (range 0.1–8.0 years).

**Figure 2.**
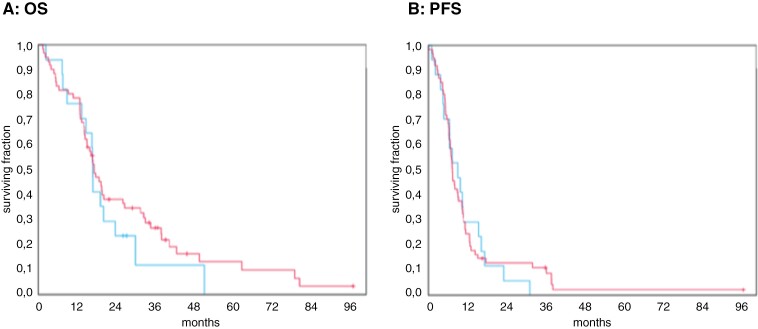
Survival outcome of patients with antibiotic use (blue) compared to no antibiotic use (red). **(A)** Median overall survival was similar in both groups (16.7 vs. 17.1 months, *P* = .404) Multivariable regression analysis showed a trend towards decreased survival in patients treated with antibiotics (HR 1.92, 95% CI: 0.91–4.08). **(B)**Median progression-free survival was similar in both groups (9.0 vs. 7.4 months, *P* = .844). No difference was observed in multivariable regression analysis (HR 1.04, 95% CI: 0.53–2.01).

Together, 66 of the 78 patients (84.6 %) died within the observation period. The median survival time was 16.7 months (95% CI: 14.7–18.7). The 1-, 2- and 5-year survival rates were 78.2%, 34.8%, and 10.3%, respectively. Patients who received antibiotics had a median survival time of 16.7 months, compared to 17.1 months for the group that did not receive antibiotics (*P* = .404). The univariable regression analysis showed no significant difference between the 2 groups. Our multivariable analysis showed a trend towards worse OS with a hazard ratio of 1.92 (95% CI: 0.91–4.08; *P* = .089) in patients with prediagnostic antibiotics (Figure 2).

We also stratified survival based on the presence or absence of lymphocytopenia. Median overall survival was similar in both groups (18.9 vs. 15.7 months, *P* = .606). Multivariable regression analysis showed no significant difference between these groups (HR 0.47, 95% CI: 0.19–1.17 *P* = .106).

In total, 75 of the 78 patients (96.1 %) showed progressive disease within the observation period. The median PFS time was 7.4 months (95% CI: 6.29–8.42). The 1-, 2- and 5-year PFS rates were 25.6%, 11.2%, and 1.7 %, respectively. Patients who received antibiotics had a median PFS time of 9.0 months, compared to 7.4 months for the group that did not receive antibiotics (*P* = .844). Furthermore, multivariable Cox regression analysis revealed no significant difference in PFS with a hazard ratio of 1.04 (95% CI: 0.53–2.01; *P* = .91) in patients with prediagnostic antibiotics (Figure 2).

## Discussion

Here, we present results from the first study that investigated possible associations between antibiotic use and the occurrence of radiochemotherapy-related toxicities in patients with GB. Antibiotics can disrupt the balance of beneficial and harmful bacteria, leading to dysbiosis and diminished levels of bacterial diversity.^33^ Dysbiosis has been linked to the regulation of immune response,^34^ which could affect how a patient responds to chemotherapy.^15^ According to this, antibiotic-pretreated germ-free glioma mice showed strongly increased tumor growth, presumably due to the reduction of cytotoxic natural killer cells, as well as microglial changes towards a protumoral and immunosuppressive phenotype.^26^ Another hypothesis addresses alterations of systemically available metabolites in dependence on microbial composition^35^ as short-chain fatty acids like butyrate have been shown to affect the response and toxicity of chemotherapy.^35^

In our analysis, prediagnostic antibiotics have been proven to be a significant predictor for reported side effects in general, and particularly for nausea and myelosuppression in multivariable models. The influence of antibiotics on overall side effects has been documented in other types of cancer.^18,20^ In the context of metastatic pancreatic adenocarcinoma, one study observed a crucial role of antibiotics in modifying the toxicity induced by gemcitabine,^20^ which is in accordance with our results. Also, antibacterial use leads to an increased risk of hematologic toxicity and gastrointestinal dose-limiting side effects of chemotherapy via the reduction of the isoform population of cytidine deaminase which is relevant to gemcitabine metabolism (CDD).^20^ Lastly, in patients with melanoma, the use of antibiotics increased the risk of colitis during immunotherapy.^18^ This could be due to the reduction of microbiome diversity and the high presence of pro-inflammatory bacteria following the use of antimicrobial drugs.^18^

Nausea was observed more often in patients who received antibiotic drugs before the time of diagnosis. The intestinal microbiota was shown to play a role in the modulation of chemotherapy-induced nausea in mice by inhibition of serotonin and neurokinin receptors.^36^ This delineates a possible explanation for this side effect in our cohort involving dysbiosis, observed after antibiotic treatment.^33,37^

We also observed a higher risk for lymphocytopenia based on laboratory values anytime during the course of treatment after prediagnostic use of antibiotics. This is in accordance with a study in patients with soft tissue and bone sarcoma, which found a correlation between antibiotic use and lymphocytopenia that was negatively associated with outcome.^38^ The authors hypothesized that antibiotics could disrupt the autoregulation of pro-inflammatory and anti-inflammatory molecules, thereby adversely impacting antitumor immunity.^38^

We found that prediagnostic antibiotic use was an independent predictor for increased CRP levels in the first week after resection. Among other factors, CRP levels can rise following craniotomy.^39^ However, even short-term use of antibiotics may result in enduring changes to the intestinal microbiota.^37,40^ There is increasing evidence that these changes are associated with the downregulation of parts of the innate immune system such as C-type lectin which is important for the immune defense against gram-positive bacteria.^40^ Therefore, it is conceivable that patients treated with antibiotics are more prone to infection, resulting in higher CRP levels that may be predictive for prognosis in GB patients.^41^

Finally, we observed a trend toward worse survival in patients who received antibiotics before the time of diagnosis. In our analysis cohort, we observed a median OS of 16.7 months which is in accordance with current clinical trials^2,5^ and therefore demonstrates the comparability with other cohorts. Some antibiotics have been identified to directly interact with chemotherapeutic drugs, which could potentially affect their metabolism and distribution throughout the body.^12–14^ This could potentially modify the effectiveness of the chemotherapy and heighten its toxicity.^13,14^ This mechanism is particularly plausible in patients undergoing treatment with temozolomide like in our cohort, as this drug is administered orally^7,42^ and therefore directly interacts with intestinal microorganisms. A xenograft mice model showed that antibiotic use affected not only glioma growth but also reduced the effectiveness of temozolomide.^27^ This was mediated through immunomodulation, which resulted in an increase of macrophages and cytotoxic lymphocytes in the brain tissue.^27^ Another hypothesis is that hematologic adverse effects, such as lymphocytopenia, may heighten susceptibility to opportunistic infections. This could potentially explain the poorer outcomes observed in certain patients.^38^ Together, our and other data, discussed above, suggest that prediagnostic antibiotic use could have a substantial impact on patient outcomes. In contrast, a recent national cohort study conducted in Sweden observed no association between antibiotic use and survival in patients with colorectal cancer.^43^ Nevertheless, patients subjected to extensive antibiotic usage exhibited poorer outcomes. Prolonged antibiotic administration induces dysbiosis within the gut microbiome, consequently precipitating anastomotic insufficiency, thus fostering additional infection.^43^ A comprehensive cohort study uncovered a notable correlation between the prescription of antibiotics before diagnosis and the overall survival rates of patients with various types of cancer. These included leukemia, melanoma, lymphoma, myeloma, uterine, bladder, breast, ovarian, and colorectal cancers.^44^ The findings suggest that antibiotics play a pivotal role in modulating the effectiveness of chemo- or immunotherapy through the altering immune response, enzymatic deactivation of cytostatic agents, and mucosal barrier function.^44^ Even if we observed only a similar trend in our cohort with patients with GB, we speculate that higher patient numbers would lead to statistically significant results.

Potential limitations of the present study are its retrospective design with a possible selection bias. However, patients were registered sequentially, and selection criteria were predefined and equally applied to the initial cohort. Initially, we intended to conduct subgroup analyses on antibiotic substances and groups. Given the small number of patients with different antibiotic substances (Supplementary Table S2), we were not able to consider details such as antibiotic dosage or variations of different substances, because these analyses would not produce reliable results. However, as mechanisms and targeted microorganisms vary across different antibiotics, this constrains the interpretability of our data.^45^ It is crucial for follow-up investigations or future studies by others to include larger cohorts and assess detailed data on antibiotic usage. The retrospective design of the study may also lead to possible bias, particularly regarding the investigation of survival in relation to antibiotic administration, due to unbalanced comparison groups. However, we also implemented a multivariable model to control for possible confounders. In addition, a direct measurement of microbiota was not possible due to the retrospective design of the study. Therefore, we are not able to prove the involvement of the microbiota in the associations.

Our study also has several strengths. This is the first study that focuses on the influence of antibiotics on active therapy phase-related toxicity in GB. Our initial cohort consisted of 1448 patients. From there, we applied strict selection criteria to get a highly homogeneous final cohort with high-quality data that were provided from certified or accredited databases at the cancer registry and institutional databases. The patient’s tumor data were also systematically collected from institutional databases and data on antibiotic use was obtained from internal files or GPs, based on patient records. This renders the risk of possible recall bias insignificant.

Summarized, our results demonstrate a possible link between the use of antibiotics, symptom load, and laboratory value-associated toxicity, which occurs during the active treatment phase of patients with GB, IDHwt (CNS WHO grade 4). As the prevalence of antibiotic drug prescriptions is high not only in patients with GB,^31^ but also in the general cancer population and individuals without cancer^46,47^ our results may lead to relevant hypotheses that can be investigated in future studies: The underlying mechanisms remain unclear. An attribution to changes in the intestinal microbiota cannot be made due to the lack of microbial markers in our study.

We conclude that our results should be replicated in larger well-annotated and prospectively collected cohorts that should also include sampling of intestinal microbiota. If verified, modulation of the microbiota by probiotics could play an important role in future supportive therapy.

## Supplementary Material

vdae170_suppl_Supplementary_Materials
