# Supplementary material for: Effect of antibiotic drug use on outcome and therapy-related toxicity in patients with glioblastoma—A retrospective cohort study
Source: Neurooncol Adv. 2024 Oct 4;6(1):vdae170. doi: 10.1093/noajnl/vdae170 (PMC11528512; doi:10.1093/noajnl/vdae170)
Supplement: vdae170_suppl_Supplementary_Materials [file vdae170_suppl_supplementary_materials.docx]

Supplements

Table S1 Number of prediagnostic antibiotic treatments

|  | | Number of Treatments | n | % |
| --- | --- | --- | --- | --- |
| Prediagnostic antibiotic use | 0 | | 61 | 78.2 |
|  | 1 | | 10 | 12.8 |
|  | 2 | | 4 | 5.1 |
|  | 3 | | 3 | 3.8 |
|  | total | | 78 | 100.0 |

Table S2 Absolute and relative frequencies of the reported antibiotic substances and groups

|  | yes | | no | |
| --- | --- | --- | --- | --- |
|  | n | % | n | % |
| Prediagnostic antibiotic substances |  |  |  |  |
| Penicilline | 5 | 6.4 | 73 | 93.6 |
| Cephalosporin | 1 | 1.3 | 77 | 98.7 |
| Carbapenem | 0 | 0.0 | 78 | 100.0 |
| Chinolone | 3 | 3.8 | 75 | 96.2 |
| Macrolide | 4 | 5.1 | 74 | 94.9 |
| Glycopeptide | 0 | 0.0 | 78 | 100.0 |
| Aminoglycoside | 0 | 0.0 | 78 | 100.0 |
| Tetracycline | 2 | 2.6 | 76 | 97.4 |
| Other Antibiotics | 2 | 2.6 | 76 | 97.4 |
| Prediagnostic antibiotic groups |  |  |  |  |
| Inhibition of cell wall synthesis | 6 | 7.6 | 72 | 92.3 |
| Interference with bacterial DNA or RNA | 4 | 5.1 | 74 | 94.9 |
| Inhibition of bacterial protein biosynthesis | 7 | 9.0 | 71 | 91.0 |

Table S3 Adverse events in patients with prediagnostic antibiotic use compared to no use according to CTC AE 5.0 - Sensitivity analysis: adjustment for variables with a Chi square p value lower than 0.200:

| Prediagnostic antibiotic use | | | | | |  | Binary logistic regression | | | | |
| --- | --- | --- | --- | --- | --- | --- | --- | --- | --- | --- | --- |
|  | yes (n=17) | | no (n=61) | | Chi^2^  *p* |  | univariable | | | multivariable*** | |
|  | n | % | n | % |  |  | p | OR (95% CI) |  | p | OR (95% CI) |
| Any side effect | 15 | 88.2 | 42 | 68.9 | 0.111 |  | 0.128 | 3.39 (0.71-16.34) | | 0.029 | 90.8 (1.26-65.34) |
| Myelosuppression | 7 | 41.2 | 11 | 18.0 | 0.045 |  | 0.052 | 3.18 (0.99-10.21) | | 0.026 | 5.59 (1.23-25.43) |
| Inflammation | 0 | 0.0 | 4 | 6.6 | 0.278 |  | - | - | | - | - |
| Skin reaction | 0 | 0.0 | 7 | 11.5 | 0.143 |  | - | - | | - | - |
| Nausea | 9 | 52.9 | 17 | 27.9 | 0.052 |  | 0.058 | 2.91 (0.97-8.79) | | 0.026 | 4.74 (1.20-18.67) |
| Vomiting | 1 | 5.9 | 8 | 13.1 | 0.409 |  | 0.422 | 0.41 (0.05-3.56) | | 0.672 | 0.57 (0.04-7.63) |
| Diarrhea | 1 | 5.9 | 2 | 3.3 | 0.622 |  | 0.626 | 1.84 (0.16-21.65) | | 0.420 | 19.61(0.01-27252.83) |
| Constipation | 1 | 5.9 | 0 | 0.0 | 0.057 |  | - | - | | - | - |
| Loss of appetite | 4 | 23.5 | 3 | 4.9 | 0.018 |  | 0.030 | 5.95 (1.19-29.86) | | 0.038 | 13.08 (1.16-148.03) |
| Dizziness. | 2 | 11.8 | 10 | 16.4 | 0.640 |  | 0.642 | 0.68 (0.13-3.45) | | 0.811 | 0.80 (0.13-5.02) |
| Headache | 2 | 11.8 | 10 | 16.4 | 0.640 |  | 0.642 | 0.68 (0.13-3.45) | | 0.946 | 0.93 (0.13-6.60) |
| Fatigue | 10 | 58.8 | 31 | 50.8 | 0.559 |  | 0.560 | 1.38 (0.47-4.11) | | 0.257 | 2.13 (0.58-7.92) |
| Alopecia | 4 | 23.5 | 9 | 14.8 | 0.391 |  | 0.395 | 1.78 (0.47-0.69) | | 0.138 | 3.90 (0.65-23.52) |

* Adjustement for the following variables: age, previous independent tumor disease

Table S4 Adverse events in patients with pretherapeutic antibiotic use compared to no use according to CTC AE 5.0

| pretherapeutic antibiotic use | | | | | |  | Binary logistic regression | | | | |
| --- | --- | --- | --- | --- | --- | --- | --- | --- | --- | --- | --- |
|  | yes (n=19) | | no (n=59) | | Chi^2^  *p* |  | univariable | | | multivariable | |
|  | n | % | n | % |  |  | p | OR (95% CI) |  | p | OR (95% CI) |
| Any side effect | 17 | 89.5 | 40 | 67.8 | 0.064 |  | 0.080 | 4.04 (0.85-19.28) | | 0.019 | 20.89 (1.64-266.68) |
| Myelosuppression | 8 | 42.1 | 10 | 16.9 | 0.024 |  | 0.028 | 3.56 (1.14-11.11) | | 0.010 | 11.70 (1.80-75.92) |
| Inflammation | 0 | 0.0 | 4 | 6.8 | 0.244 |  | * | * | | * | * |
| Skin reaction | 1 | 5.3 | 6 | 10.2 | 0.515 |  | 0.523 | 0.49 (0.06-4.36) | | * | * |
| Nausea | 10 | 52.6 | 16 | 27.1 | 0.040 |  | 0.045 | 2.99 (1.03-8.69) | | 0.061 | 5.21 (0.93-29.36) |
| Vomiting | 2 | 10.5 | 7 | 11.9 | 0.874 |  | 0.874 | 0.87 (0.17-4.62) | | 0.742 | 0.61 (0.03-12.0) |
| Diarrhea | 1 | 5.3 | 2 | 3.4 | 0.712 |  | 0.714 | 1.58 (0.14-18.50) | | * | * |
| Constipation | 1 | 5.3 | 0 | 0.0 | 0.076 |  | * | * | | * | * |
| Loss of appetite | 5 | 26.3 | 2 | 3.4 | 0.002 |  | 0.009 | 10.18(1.79-58.04) | | 0.630 | 0.39 (0.01-18.59) |
| Dizziness. | 4 | 21.1 | 8 | 13.6 | 0.431 |  | 0.435 | 1.70 (0.45-6.44) | | 0.132 | 5.70 (0.60-54.63) |
| Headache | 2 | 10.5 | 10 | 16.9 | 0.500 |  | 0.504 | 0.58 (0.12-2.90) | | 0.671 | 0.59 (0.05-6.65) |
| Fatigue | 11 | 57.9 | 30 | 50.8 | 0.593 |  | 0.593 | 1.33 (0.47-3.78) | | 0.596 | 1.45 (0.36-5.81) |
| Alopecia | 4 | 21.1 | 9 | 15.3 | 0.555 |  | 0.557 | 1.48 (0.40-5.50) | | 0.435 | 2.23 (0.30-16.57) |

Table S5 Mean values of hematologic toxicity parameters depending on prediagnostic antibiotic use vs. no use during the entire observation period

| t-Test | | | | |  | | Regression analysis | |
| --- | --- | --- | --- | --- | --- | --- | --- | --- |
|  | Prediagnostic  antibiotic use | N | mean | *p* |  | univariable | | multivariable |
|  |  |  |  |  |  | p | | p |
| Leukocytes  (mio/ml) | yes | 16 | 5.91 | 0.441 |  | 0.813 | | 0.122 |
|  | no | 54 | 6.17 |  |  |  |  |  |
| Thrombocytes  (mio/ml) | yes | 16 | 145 0000 | 0.857 |  | 0.108 | | 0.001 |
|  | no | 54 | 175 4259 |  |  |  |  |  |
| Lymphocytes  (mio/ml) | yes | 11 | 0.6583 | 0.061 |  | 0.093 | | 0.574 |
|  | no | 41 | 0.9962 |  |  |  |  |  |
| Granulocytes  (mio/ml) | yes | 11 | 3.0707 | 0.062 |  | 0.286 | | 0.900 |
|  | no | 41 | 4.0110 |  |  |  |  |  |
| CRP  (mg/dl) | yes | 15 | 30.13 | 0.417 |  | 0.528 | | 0.630 |
|  | no | 50 | 19.62 |  |  |  |  |  |

Table S6 Hematologic laboratory parameters in patients with prediagnostic antibiotic use compared to no use in week 1, 12, 20, 28 +/- 2 after time of diagnosis

| Laboratory alterations | | Prediagnostic antibiotic use | | | | |  | Binary logistic regression | | | |
| --- | --- | --- | --- | --- | --- | --- | --- | --- | --- | --- | --- |
|  |  | yes | | no | | *p* |  | univariable | | multivariable** | |
|  |  | n | % | n | % |  |  | p | OR (95% CI) | p | OR (95% CI) |
| Week 1 | | | | | | |  |  | | | |
| Leucocytes | CTC 0-2  CTC 3-4 | 15  0 | 100.0  0.0 | 45  0 | 100.0  0.0 | - |  | - | 0.34 (0.01-17.91) | - | - |
| Lymphocytes | CTC 0-2  CTC 3-4 | 2  3 | 40.0  60.0 | 13  3 | 81.3  18.8 | 0,075 |  | 0.093 | 6.50 (0.73-57.83) | - | - |
| Granulocytes | CTC 0-2  CTC 3-4 | 5  0 | 100.0  0.0 | 16  0 | 100.0.  0.0 | - |  | - | 0.33 (0.01-18.88) | - | - |
| Thrombocytes | CTC 0-2  CTC 3-4 | 15  0 | 100.0  0.0 | 46  0 | 100.0  0.0 | - |  | - | 0.33 (0.01-17.52) | - | - |
| Hemoglobin | CTC 0-2  CTC 3-4 | 15  0 | 100.0  0.0 | 46  0 | 100.0  0.0 | - |  | - | 0.33 (0.01-17.52) | - | - |
| CRP (mg/l) | < 5,0  ≥ 5,0 | 4  3 | 57.1  42.9 | 26  11 | 70.3  29.7 | 0,494 |  | 0.104 | 3.06 (0.80-11.76) | 0.021 | 20.858 (1.57-277.00) |
| Week 12 | | | | | |  |  |  | |  |  |
| Leucocytes | CTC 0-2  CTC 3-4 | 11  0 | 100.0  0.0 | 38  1 | 97.4  2.6 | 0.592 |  | 0.999 | 0.9 (0.03-23.52) | - | - |
| Lymphocytes | CTC 0-2  CTC 3-4 | 4  5 | 44.4  55.5 | 13  14 | 48.1  51.9 | 0,847 |  | 0.847 | 1.61 (0.26-5.29) | 0.36 | 2.93 (0.29-29.94) |
| Granulocytes | CTC 0-2  CTC 3-4 | 9  0 | 100.0  0.0 | 26  1 | 96.3  3.7 | 0.558 |  | 0.999 | 1.08 (0.04-28.73) | 1.00 | 1.573 |
| Thrombocytes | CTC 0-2  CTC 3-4 | 10  1 | 90.9  9.1 | 39  0 | 100.0  0.0 | 0.057 |  | 0.999 | 0.09 (0.00-2.34) | - | - |
| Hemoglobin | CTC 0-2  CTC 3-4 | 11  0 | 100.0  0.0 | 38  1 | 97.4  2.6 | 0.592 |  | 0.999 | 0.9 (0.03-23.52) | 1.00 | 0.235 |
| CRP (mg/l) | < 5,0  ≥ 5,0 | 9  0 | 100.0  0 | 22  4 | 84.6  15.5 | 0.211 |  | 0.209 | 0.24 (0.03-2.20) | 0.16 | 0.06 (0.00-3.08) |
| Week 20 | | | | | | |  |  | |  | |
| Leucocytes | CTC 0-2  CTC 3-4 | 9  1 | 90.0  10.0 | 24  1 | 96.0  4.0 | 0.490 |  | 0.504 | 2.67 (0.15-47.30) | - | - |
| Lymphocytes | CTC 0-2  CTC 3-4 | 2  5 | 28.6  71.4 | 5  11 | 31.3  68.8 | 0,898 |  | 0.089 | 1.14 (0.16-8.00) | - | - |
| Granulocytes | CTC 0-2  CTC 3-4 | 6  1 | 85.7  14.3 | 14  2 | 87.5  12.5 | 0.907 |  | 0.907 | 1.16 (0.08-15.46) | - | - |
| Thrombocytes | CTC 0-2  CTC 3-4 | 8  1 | 88.9  11.1 | 24  1 | 96.0  4.0 | 0.437 |  | 0.455 | 3.00 (0.17-53.71) | - | - |
| Hemoglobin | CTC 0-2  CTC 3-4 | 9  0 | 100.0  0.0 | 24  1 | 96.0  4.0 | 0.543 |  | 0.999 | 1.16 (0.04-31-14) | - | - |
| CRP (mg/l) | < 5,0  ≥ 5,0 | 3  2 | 60.0  40.0 | 14  2 | 87.5  12.5 | 0,172 |  | 0,137 | 4,67 (0.61-35.49) | - | - |
| Week 28 | | | | | | |  |  | |  | |
| Leucocytes | CTC 0-2  CTC 3-4 | 11  0 | 100.0  0.0 | 29  2 | 93.5  6.5 | 0.388 |  | 0.999 | 1.95 (0.09-43.78) | - | - |
| Lymphocytes | CTC 0-2  CTC 3-4 | 2  6 | 25.0  75.0 | 7  14 | 33.3  66.7 | 0.665 |  | 0,666 | 1,50 (0.24-9.44) | - | - |
| Granulocytes | CTC 0-2  CTC 3-4 | 8  0 | 100.0  0.0 | 19  2 | 90.5  9.5 | 0.366 |  | 0.999 | 2.18 (0.09-50.43) | - | - |
| Thrombocytes | CTC 0-2  CTC 3-4 | 11  0 | 100.0  0.0 | 29  2 | 93.5  6.5 | 0.388 |  | 0.999 | 1.95 (0.09-43.78) | - | - |
| Hemoglobin | CTC 0-2  CTC 3-4 | 11  0 | 100.0  0.0 | 30  1 | 96.8  3.2 | 0.547 |  | 0.999 | 1.13 (0.04-29.81) | - | - |
| CRP (mg/l) | < 5,0  ≥ 5,0 | 4  2 | 66.7  33.3 | 17  1 | 94.4  5.6 | 0.075 |  | 0,464 | 2,13 (0.28-15.97) | - | - |

* low case numbers

** Adjustement for the following variables: Sex, age, BMI, MGMT, Karnofsky score, extent of resection, previous independent tumor disease
